# A Comparison of Shiga-Toxin 2 Bacteriophage from Classical Enterohemorrhagic *Escherichia coli* Serotypes and the German *E. coli* O104:H4 Outbreak Strain

**DOI:** 10.1371/journal.pone.0037362

**Published:** 2012-05-23

**Authors:** Chad R. Laing, Yongxiang Zhang, Matthew W. Gilmour, Vanessa Allen, Roger Johnson, James E. Thomas, Victor P. J. Gannon

**Affiliations:** 1 Laboratory for Foodborne Zoonoses, Public Health Agency of Canada, Lethbridge, Alberta, Canada; 2 National Microbiology Laboratory, Public Health Agency of Canada, Winnipeg, Manitoba, Canada; 3 Ontario Agency for Health Protection and Promotion, Ontario, Canada; 4 Laboratory for Foodborne Zoonoses, Public Health Agency of Canada, Guelph, Ontario, Canada; 5 Department of Biological Sciences, University of Lethbridge, Lethbridge, Alberta, Canada; U. S. Salinity Lab, United States of America

## Abstract

*Escherichia coli* O104:H4 was associated with a severe foodborne disease outbreak originating in Germany in May 2011. More than 4000 illnesses and 50 deaths were reported. The outbreak strain was a typical enteroaggregative *E. coli* (EAEC) that acquired an antibiotic resistance plasmid and a Shiga-toxin 2 (Stx2)-encoding bacteriophage. Based on whole-genome phylogenies, the O104:H4 strain was most closely related to other EAEC strains; however, Stx2-bacteriophage are mobile, and do not necessarily share an evolutionary history with their bacterial host. In this study, we analyzed Stx2-bacteriophage from the *E. coli* O104:H4 outbreak isolates and compared them to all available Stx2-bacteriophage sequences. We also compared Stx2 production by an *E. coli* O104:H4 outbreak-associated isolate (ON-2011) to that of *E. coli* O157:H7 strains EDL933 and Sakai. Among the *E. coli* Stx2-phage sequences studied, that from O111:H- strain JB1-95 was most closely related phylogenetically to the Stx2-phage from the O104:H4 outbreak isolates. The phylogeny of most other Stx2-phage was largely concordant with their bacterial host genomes. Finally, O104:H4 strain ON-2011 produced less Stx2 than *E. coli* O157:H7 strains EDL933 and Sakai in culture; however, when mitomycin C was added, ON-2011 produced significantly more toxin than the *E. coli* O157:H7 strains. The Stx2-phage from the *E. coli* O104:H4 outbreak strain and the Stx2-phage from O111:H- strain JB1-95 likely share a common ancestor. Incongruence between the phylogenies of the Stx2-phage and their host genomes suggest the recent Stx2-phage acquisition by *E. coli* O104:H4. The increase in Stx2-production by ON-2011 following mitomycin C treatment may or may not be related to the high rates of hemolytic uremic syndrome associated with the German outbreak strain. Further studies are required to determine whether the elevated Stx2-production levels are due to bacteriophage or *E. coli* O104:H4 host related factors.

## Introduction

A novel *Escherichia coli* O104:H4 strain was associated with a widespread and severe foodborne disease outbreak in Germany between early May and July, 2011 [1]. Among the more than 4000 individuals from which the pathogen was isolated, approximately 800 people developed the life-threatening hemolytic uremic syndrome (HUS) and 50 people succumbed to the illness. Epidemiological studies eventually pointed to a single lot of imported Fenugreek seeds used to prepare sprouts for salads as the source of the organism [2].

The *E. coli* O104:H4 outbreak strain exhibited characteristics typical of other enteroaggregative *E. coli* (EAEC), including the presence of a paa-containing virulence plasmid, production of enteroaggregative adherence fimbriae (AAF) I, and formation of the stacked-brick adherence pattern on intestinal epithelial cells [3]. The genome of the outbreak strain was also very similar to the EAEC O104:H4 reference strain 55989, which was isolated in Africa in 2002 [4]. The 2011 outbreak isolates were shown to be clonal and part of multi-locus sequence type ST678, which is unique to *E. coli* O104:H4 strains, and part of the *E. coli* phylogenetic group B1, which contains a variety of other pathogenic serotypes [5]. The outbreak strains also acquired an antibiotic resistance plasmid, conferring resistance to all penicillins, cephalosporins and co-trimoxazole [3], [6]. Unusually, the outbreak-associated *E. coli* O104:H4 isolates also produced Shiga-toxin 2 (Stx2) and consequently were classified as Shiga-toxin producing *E. coli* (STEC). Stx production has only rarely been reported among EAEC strains. Prior to the outbreak, only seven sporadic cases of infections with *E. coli* O104:H4 strains that produced Stx2 had been reported worldwide in the preceding ten years [7].

Prior to this outbreak, HUS in Germany and elsewhere was most frequently associated with STEC infections in children and the elderly, and only 1.5%–10% of all reported STEC cases in Germany between 2006 and 2010 resulted in HUS in adults [8]. By contrast, in the *E. coli* O104:H4 outbreak, illness occurred predominantly in otherwise healthy adults and approximately 20% of the cases resulted in HUS, and severe neurological complications were also observed in a large number of cases [3], [9]. *E. coli* O104:H4 had previously been associated with only two cases of HUS; one in a woman from Tokyo and one from a child in Germany [3].

STEC can harbour one or more *stx*-genes which are encoded by inducible lambda phage integrated into their genomes, and the entire phage and specific regions within the phage can be gained or lost through horizontal gene transfer [10]. The *E. coli* O104:H4 outbreak isolates contained two integrated Stx-like phages, one of which contained the Stx2 A and B subunit genes and another that did not [5].

Whole-genome phylogenies place the *E. coli* O104:H4 outbreak strain as most closely related to other *E. coli* O104:H4 and EAEC strains, whether they have acquired the Stx2-phage or not [5], [11]. Because Stx-bacteriophage are mobile, their evolutionary history may differ from that of their bacterial host [12]. This raises the question as to the possible origin of the Stx2-phage found in the *E. coli* O104:H4 outbreak strain.

In this study, we analyzed the Stx2-phage sequences from the *E. coli* O104:H4 outbreak strain and compared it among 51 other Stx2-phage sequences from Genbank and those obtained using *de novo* DNA sequence analysis. We demonstrate that the *E. coli* O104:H4 Stx2-phage is most closely related to the Stx2-phage from *E. coli* O111:H- strain JB1-95, and that a recent common ancestor likely gave rise to both it and the phage present in the outbreak strain. Additionally we show that Stx2 production by an *E. coli* O104:H4 outbreak-related isolate is significantly greater than that of two other outbreak-related *E. coli* O157:H7 strains following addition of mitomycin-C to cultures.

## Materials and Methods

### Identification and Isolation of Stx2-bacteriophage Sequences

The Stx2 subunit A nucleotide sequence from *E. coli* O157:H7 strain EDL933 (NC_002655.2 1352290..1353249) was used to identify Stx2-bacteriophage containing bacteria within Genbank, both the closed and whole-genome shotgun sequence databases. These sequences were downloaded and the Stx2-bacteriophage sequence was identified using PHAST [13]. Only continuous phage sequences of ≥20 kb from a single contig or closed-genome were used in subsequent analyses; Stx2-positive draft genomes that did not meet these criteria were excluded. The Stx1-like phage from *Shigella dysenteriae* was included as an outgroup for the analyses. The names, sources and accession numbers of all 52 bacteriophage sequences used in this study are presented in Table 1.

**Table 1:** The Stx2-bacteriophage and genomic sequences used in the current study.

Genbank ID	Bacterial Isolate	Bacteriophage	Source
ABHK	O157:H7 EC4206	Stx2, Stx2c	J. Craig Venter Institute
ABHM	O157:H7 EC4042	Stx2, Stx2c	J. Craig Venter Institute
ABHO	O157:H7 EC4196	Stx2	J. Craig Venter Institute
ABHP	O157:H7 EC4113	Stx2	J. Craig Venter Institute
ABHQ	O157:H7 EC4076	Stx2	J. Craig Venter Institute
ABHR	O157:H7 EC4401	Stx2	J. Craig Venter Institute
ABHS	O157:H7 EC4486	Stx2	J. Craig Venter Institute
ABHU	O157:H7 EC869	Stx2c	J. Craig Venter Institute
ABHW	O157:H7 EC508	Stx2	J. Craig Venter Institute
ABJT	O157:H7 EC4024	Stx2	J. Craig Venter Institute
ABKY	O157:H7 TW14588	Stx2, Stx2	J. Craig Venter Institute
ADUQ	O111:NM OK1180	Stx2	[47]
AE005174.2	O157:H7 EDL933	Stx2	[20]
AERP	O157:H7 1044	Stx2	Virginia Bioinformatics Institute
AERQ	O157:H7 EC1212	Stx2c	Virginia Bioinformatics Institute
AERR	O157:H7 1125 ECF	Stx2	Los Alamos National Lab
AEZV	O111:H- JB1-95	Stx2	J. Craig Venter Institute
AEZX	O121:H19 5.0959	Stx2	J. Craig Venter Institute
AEZZ	O128:H2 9.0111	Stx2	J. Craig Venter Institute
AF125520.1	n/a	Bacteriophage 933****W	[48]
AFAA	O145:H2 4.0967	Stx2	J. Craig Venter Institute
AFAB	O147:H- 2.3916	Stx2e	J. Craig Venter Institute
AFAC	O153:H- 3.3884	Stx2	J. Craig Venter Institute
AFDQ	O91:H21 B2F1	Stx2	University of Maryland, IGS
AFDR	O73:H16 C165-02	Stx2d	University of Maryland, IGS
AFDW	Ont:H12 EH250	Stx2d	University of Maryland, IGS
AFEA	O139 S1191	Stx2e	University of Maryland, IGS
AFOG	O104:H4 TY-2482	Stx2	[49]
AFST	O104:H4 C227-11	Stx2	[45]
AFWB	O104:H4 CS110	Stx2	Health Protection Agency, UK
AFWC	O104:H4 CS70	Stx2	Health Protection Agency, UK
AFWO	O104:H4 GOS1	Stx2	[5]
AFWP	O104:H4 GOS2	Stx2	[5]
AHZF	O104:H4 ON-2011	Stx2	[21]
AP005154.1	n/a	Bacteriophage Stx2_II	[50]
AP010958.1	O103:H2 12009	Stx2	[47]
AP010960.1	O111:H- 11128	Stx2	[47]
BA000007.2	O157:H7 Sakai	Stx2	[19]
CP001164.1	O157:H7 EC4115	Stx2, Stx2c	[51]
CP001368.1	O157:H7 TW14359	Stx2, Stx2c	[52]
CP002890.1	O147 UMNF18	Stx2e	[53]
FM180578.1	n/a	Bacteriophage 2851	[54]
HQ424691.1	O157:H7 71074	Stx2	Public Health Agency of Canada
in-house	O157:H7 LRH6	Stx2	Public Health Agency of Canada
in-house	O157:H7 EC970520	Stx2c	Public Health Agency of Canada
NC_003525.1	n/a	Bacteriophage Stx2_I	[55]
NC_007606.1	*Shigella dysenteriae* Sd197	Stx1	[56]

### Phylogenetic Relationships Among Stx2-bacteriophage

A non-redundant Stx2-bacteriophage “pan-genome” of the 52 phage-genomes of this study was created, and fragments ≥500 bp present in at least three of the Stx2-phage genomes were aligned using the standalone-version of the Panseq program (http://lfz.corefacility.ca/panseq/) with the following settings: ‘minimumNovelRegionSize’ = >‘500’, fragmentationSize’ = >‘500’, ‘nucB’ = >‘200’, ‘nucC’ = >‘50’, ‘nucD’ = >‘0.12’, ‘nucG’ = >‘100’, ‘nucL’ = >‘11’, ‘percentIdentityCutoff’ = >‘85’, ‘coreGenomeThreshold’ = >‘3’ [14]. The aligned fragments were visualized in SplitsTree 4 (http://www.splitstree.org/), using the uncorrected P distance and the neighbor-net algorithm [15]. We have previously shown these fragmentation and sequence identity threshold parameters to be appropriate choices for *E. coli* [14]. Figure S1 depicts the neighbor-nets of the same data fragmented from 50 bp –1000 bp at both an 85% and 95% sequence identity cutoff, all of which show similar relationships among strain groups.

A maximum-likelihood tree using the aligned Stx2-bacteriophage “pan-genome” described above was created using PhyML 3.0 with the following settings: PhyML_3.0_linux64 -p -m HKY85 -s BEST –rand_start –n_rand_starts 5–r_seed 5 [16]. The resulting tree file with approximate likelihood ratio test (aLRT) branch support values was visualized in Dendroscope [17].

**Figure 1:**
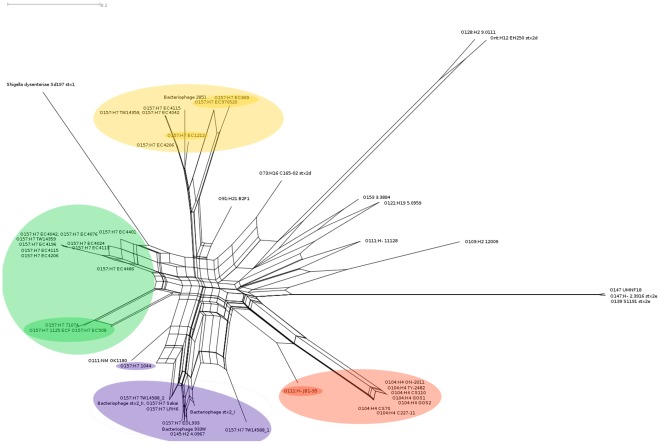
The neighbor-net visualization of the Stx2 “pan-genome” among *E. coli* Stx2-bacteriophage and a bacteriophage from *Shigella dysenteriae* (**Table 1**). The “pan-genome” consists of 500 bp sequence fragments present in at least 3 bacteriophage sequences at an 85% sequence identity threshold. Labels represent the host-source of the bacteriophage as serotype followed by strain name. Red shading: Stx2-phage from O104:H4 outbreak isolates (light); O111:H- strain JB1-95 (dark). Purple shading: Stx2-phage from O157:H7 lineage I strains; related Stx2-bacteriophge; O145:H2 strain 4.0967. Green shading: Stx2-phage from O157:H7 lineage I/II strains; the sub-group of lineage I/II Stx2-phage most closely related to the O104:H4 isolate phage. Yellow shading: Stx2c-phage from O157:H7 lineage I/II strains and bacteriophage 2851 (light); O157:H7 lineage II strains (dark).

**Figure 2:**
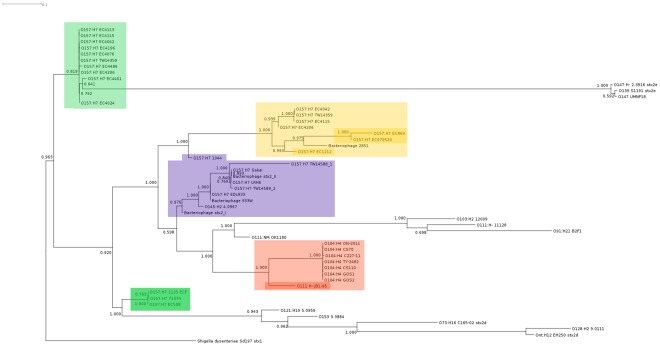
Maximum likelihood phylogram with aLRT branch-support values of the Stx2 “pan-genome” data among *E. coli* Stx2-bacteriophage and a bacteriophage from *Shigella dysenteriae* (**Table 1**). Labels represent the host-source of the bacteriophage as serotype followed by strain name. Red shading: Stx2-phage from O104:H4 outbreak isolates (light); O111:H- strain JB1-95 (dark). Purple shading: Stx2-phage from O157:H7 lineage I strains; related Stx2-bacteriophge; O145:H2 strain 4.0967. Green shading: Stx2-phage from O157:H7 lineage I/II strains; the sub-group of lineage I/II Stx2-phage most closely related to the O104:H4 isolate phage. Yellow shading: Stx2c-phage from O157:H7 lineage I/II strains and bacteriophage 2851 (light); O157:H7 lineage II strains (dark).

The Stx2-bacteriophage that were closest to the *E. coli* O104:H4 outbreak isolates in Figures 1 and 2 were aligned using MAFFT v6.857b with the command: mafft –maxiterate 1000–clustalout –thread 22 [18]. Unlike the non-syntenic Panseq alignment, the MAFFT alignment was based on uninterrupted, whole bacteriophage genomes. The MAFFT-aligned genomes were subjected to maximum likelihood phylogenetic analysis as previously described and visualized in Dendroscope [17].

### Whole-genome Phylogeny

A non-redundant “pan-genome” of 42 STEC and *Shigella dysenteriae* whole-genomes was created, and fragments ≥500 bp present in at least three of the genomes were aligned using the standalone-version of the Panseq program (http://lfz.corefacility.ca/panseq/) with the following settings: ‘minimumNovelRegionSize’ = >‘500’, fragmentationSize’ = >‘500’, ‘nucB’ = >‘200’, ‘nucC’ = >‘50’, ‘nucD’ = >‘0.12’, ‘nucG’ = >‘100’, ‘nucL’ = >‘20’, ‘percentIdentityCutoff’ = >‘85’, ‘coreGenomeThreshold’ = >‘3’ [14]. A maximum-likelihood tree with aLRT branch support values was created using PhyML as described above [16].

### Antibiotic Induction

Overnight cultures of each strain from single colonies were inoculated in 1 ml of BHI medium and incubated at 37°C with shaking at 150 rpm in 2 ml microfuge tubes for 17 hours. The overnight culture was diluted 1∶100 in fresh BHI medium and incubated at 37°C with shaking at 150 rpm for 3 hours after which optical density at 600 nm was measured. Cultures were diluted in two serial dilutions by factors of two to reach final optical densities ∼0.6. Antibiotic concentrations of 0.015625 µg/ml ciprofloxacin and 0.125 µg/ml norfloxacin were used for the induction of EDL 933; 0.0625 µg/ml ciprofloxacin and 0.125 µg/ml norfloxacin were used for the induction of Sakai; 0.0625 µg/ml ciprofloxacin and 0.25 µg/ml norfloxacin were used for the induction of ON-2011. A concentration of 0.5 µg/ml mitomycin was used for all three strains. Cultures were incubated at 37°C with shaking at 150 rpm for 18 hours, which Vareille et al. (2007) found to be critical for maximum Stx2 expression [23]. Optical density at 600 nm was measured with ten-fold dilutions of culture.

### Quantification of Stx2 Production

Three Stx2-producing strains were used for the quantification of Stx2 production; two were *E. coli* O157:H7 strains associated with large outbreaks (strains Sakai [19] and EDL933 [20]) and were obtained from the American Type Culture Collection, and one a travel-associated isolate from the German 2011 *E. coli* O104:H4 outbreak (strain ON-2011 [21]). The amount of Stx2 produced by each strain was quantified as previously described using an enzyme-linked immunosorbent assay [22], with the following modification: cells were lysed by incubation with 0.5 mg/ml polymyxin B (Sigma) at 37°C for 60 min, rather than 1.5 mg/ml for 5 min. The values of three independent experiments, each with two replicates, were used to determine the average toxin production for each strain.

### RNA Isolation

One ml of overnight culture was transferred to RNase-free 1.5 ml microfuge tubes and spun for 5 minutes at 13,000 rpm in a Legend Micro 21 R (Sorvall). Following centrifugation, 990 µl supernatant was removed, filtered using 0.20 um filters (Corning), and stored at −20°C for use in ELISA. The bacterial pellet was dissolved in 1.0 ml of RNAprotect Bacteria Reagent (Qiagen) and incubated for 5 min at room temperature (RT) and subsequently centrifuged for 10 minutes at 5000****g to remove supernatant. The pellet was re-suspended in 100 µl TE buffer (10****mM Tris:1****mM EDTA pH 8.0) with 1 mg/ml lysozyme, and incubated for 5 minutes at RT, with vortexing in 2 minute intervals. The RNeasy Mini Kit (Qiagen) was used to complete the RNA extraction and eluted RNA was stored at −80°C.

#### cDNA synthesis

Reverse transcription of isolated RNA was performed using 1 µg RNA. The genomic DNA elimination reaction consisting of 18 µl template RNA in DNase, RNase-free water (Qiagen) and 3 µl of 7× gDNA Wipeout Buffer from the QuantiTect Reverse Transcription Kit (Qiagen) was incubated for 2 minutes at 42°C in a Mastercycler pro (eppendorf). The reverse transcription reaction was conducted in a reaction mixture containing the 21 µl gDNA elimination reaction, 6 µl of 5× Quantiscript RT Buffer (Qiagen), 1.5 µl RT Primer Mix (Qiagen), and 1.5 µl Quantiscript Reverse Transcriptase. The mixture was incubated in a Mastercycler pro (Eppendorf) using a 2-stage program: 15 minutes of incubation at 42°C followed by 3 minutes at 95°C. The cDNA was stored at −20°C until needed.

### Detection of *gnd* and *stx_2_* Gene Expression by RT-PCR

RT-PCR reactions were performed using a Rotor Gene Q (Qiagen) in reaction volumes of 25 µl consisting of: 9.25 µl DNase, RNase-free water (Sigma), 12.5 µl PerfeCTa FastMix II (Quanta Biosciences), 1 µl of standard or cDNA, 7.5 pmol of each primer and probe. The probes were conjugated at the 5′ end with fluorescent reporter dyes 6-carboxyfluorescein (FAM) and proprietary fluorophore VIC for *stx2* and *gnd* probes respectively. Both probes were also conjugated at the 3′end with the Black Hole Quencher BHQ-1 (Alpha DNA). The *gnd* housekeeping gene of *E. coli* was used to normalize values between RNA samples. Standard curves were generated using three concentrations (0.1 ng/µl, 0.01 ng/µl, and 0.001 ng/µl) of plasmids pStx2R-1 and pAY01 containing *stx_2_* and *gnd* genes respectively. The RT-PCR cycling conditions used were: a hold for 2 minutes at 55°C preceding a hold for 2 minutes at 95°C and 45 cycles of 15 seconds at 95°C followed by 45 seconds at 60°C. Gain optimization was set to the first tube for both Green (FAM) and Yellow (VIC) Channels and acquisition occurred during the 60°C step in cycling. The values of three independent replicates were used to determine average mRNA copy numbers.

### Statistical Analysis

Student’s unpaired two-tailed t-test in Microsoft Excel was used to test for significance the differences between Stx2-toxin production and Stx2-mRNA production among strains.

## Results

The neighbor-net in Figure 1 depicts the Stx-phage distribution among STEC strains and *Shigella dysenteriae.* The phage in *S. dysenteriae* was most distantly related to those in the rest of the strains. The *E. coli* O157:H7 Stx2-bacteriophage were clustered based on both genetic lineage and Stx2/Stx2c-toxin sub-type. All Stx2c-bacteriophage from *E. coli* O157:H7 lineage I/II strains were from hosts that also contained a second integrated Stx2-bacteriophage. The *E. coli* O104:H4 outbreak Stx2-phage were situated as a separate cluster, nearest to the Stx2-phage from *E. coli* O111:H- strain JB1-95.

The maximum-likelihood tree of the same data used to construct Figure 1 depicts the relationship among the Stx2-phage in tree form (Figure 2). Most Stx2-phage grouped together with other Stx2-phage isolated from bacteria of the same serotype, with the exception of *E. coli* serotype O111 strains. Again, the O157:H7 Stx2-bacteriophage were clustered based on both genetic lineage and Stx2/Stx2c-toxin sub-type. The *E. coli* O104:H4 outbreak isolate Stx2-phage are most closely situated on the tree to the Stx2-phage from *E. coli* O111:H- strain JB1-95, and more distantly to Stx2-phage from other O111, O103 and O91 serotypes.

**Figure 3:**
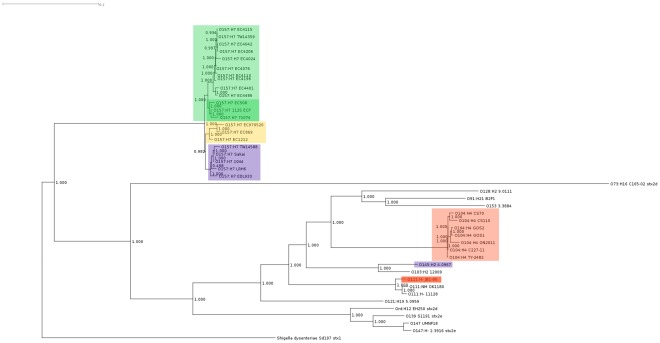
Maximum likelihood phylogram with aLRT branch-support values based on the complete pan-genome of the Stx2-containing genomic sequences in **Table 1**. Red shading: O104:H4 outbreak isolates (light); O111:H- strain JB1-95 (dark). Purple shading: O157:H7 lineage I strains; O145:H2 strain 4.0967. Green shading: O157:H7 lineage I/II strains; the sub-group of lineage I/II strains with Stx2-phage most closely related to the O104:H4 isolate phage. Yellow shading: O157:H7 lineage II strains.

As Stx2-phage are mobile, we compared the relationships among the host bacterial genomes to determine the concordance of the Stx2-phage with that of their host-genome (Figure 3). The groupings of Stx2-phage and host Stx2-genomes were surprisingly similar, with the notable exception of Stx2-phage and bacterial genomes from serogroup O111, which formed a discrete cluster in the whole-genome tree, but were more widely distributed in the Stx2-phage tree. All serotypes in the whole-genome tree of Figure 3 formed discrete clusters. The tree was divided into two separate branches, with O157:H7 strains comprising one branch, and all other STEC strains the other. Within the large O157:H7 branch of Figure 3, three sub-groups that corresponded to genetic lineages I, I/II and II were observed. O145:H2 strain 4.0967 and O103:H2 strain 12009 formed a distinct group in contrast to the Stx2-bacteriophage tree (Figure 2), where the O145:H2 strain 4.0967 Stx2-phage was part of the O157:H7 lineage I Stx2 group and O103:H2 strain 12009 Stx2-phage was most closely grouped to O111:H- strain 11128.

**Figure 4:**
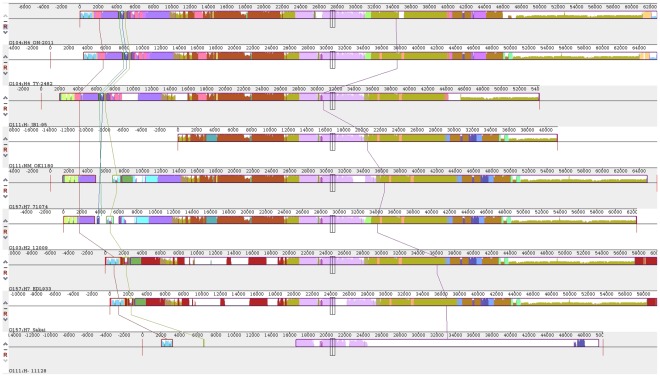
The progressiveMauve alignment of the Stx2-bacteriophage closely related to the O104:H4 outbreak isolates [**46**]. Bounded boxes indicate similar sequence composition among sequences. The black box indicates the location of the *stx2ab* gene cluster in each sequence.

**Figure 5:**
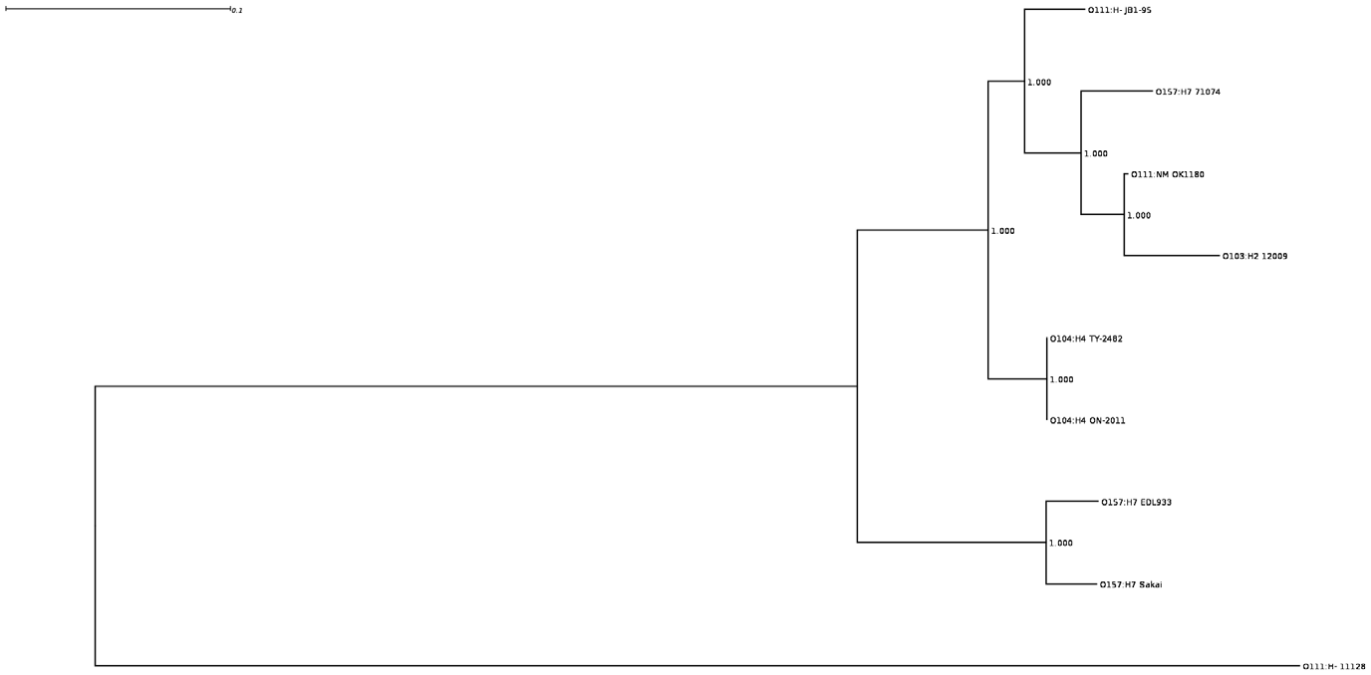
Maximum likelihood phylogram with aLRT branch-support values of the MAFFT alignment of the Stx2-bacteriophage closely related to the O104:H4 outbreak isolates.

We wished to more thoroughly examine the relationships of the entire, intact Stx2-phage sequences from those that most closely resembled the Stx2-phage from the *E. coli* O104:H4 outbreak isolates. Figure 4 shows the Mauve aligned complete Stx2-phage sequences of a selection of Stx2-bacteriophage that were closely related in Figures 1 and 2. There were common conserved regions among the strains, and Stx2-phage such as O103:H2 strain 12009 and O157:H7 71074 had what appeared to be a very similar phage structure to the O104:H4 outbreak phage, despite not being as evolutionarily conserved at the nucleotide level, as demonstrated by the phylogenetic analyses of this study. Conversely, the O111:H- strain JB1-95 Stx2-phage was grouped most-closely to the O104:H4 Stx2-phage cluster in Figures 1, 2 and 5, but showed the absence of a phage region beginning at the 45 kb region of its phage that was conserved in all the Stx2-phage except O111:H- strain 11128. The O111:H- strain 11128 Stx2-phage was the least similar to the O104:H4 outbreak Stx-2 phage among those examined in Figure 4.

Figure 5 shows the maximum likelihood phylogram of the sequences depicted in Figure 4 after MAFFT alignment; the alignment file is available as Dataset S1. This ML tree showed the *E. coli* O111:H- strain JB1-95 Stx2-bacteriophage to be most closely situated to the Stx2-bacteriophage from the German *E. coli* O104:H4 outbreak isolates, and the O111:H- strain 11128 to be most distantly related among the strains in Figure 5. The Stx2-phage from O111:H- strain JB1-95 was located on the tree between the O104:H4 outbreak cluster and a cluster of Stx2-phage from *E. coli* O157:H7 lineage I/II strain 71074, O103:H2 strain 12009, and O111:NM strain OK1180.

**Figure 6:**
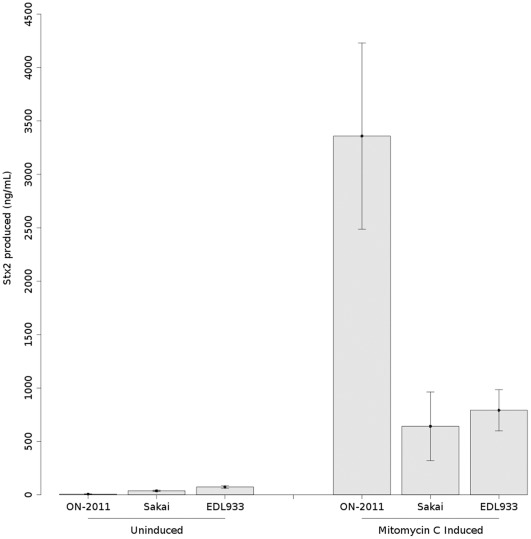
Stx2-production by *E. coli* O104:H4 outbreak-related strain ON-2011 and *E. coli* O157:H7 strains EDL933 and Sakai in un-induced, and mitomycin C-induced states as measured by a Stx2-specific ELISA. Error bars represent standard deviations from three independent replicates.

Lastly, we compared the Stx2-production by *E. coli* O104:H4 outbreak-related strain ON-2011 with that of two other outbreak–associated strains of *E. coli* O157:H7, EDL933 and Sakai. As shown in Figure 6, Stx2-production of ON-2011 was significantly lower than that of the two O157:H7 strains (Ρ <0.01). However, after the addition of mitomycin C, Stx2-production by ON-2011 was significantly greater than for both of the *E. coli* O157:H7 strains (P<0.01).

**Figure 7:**
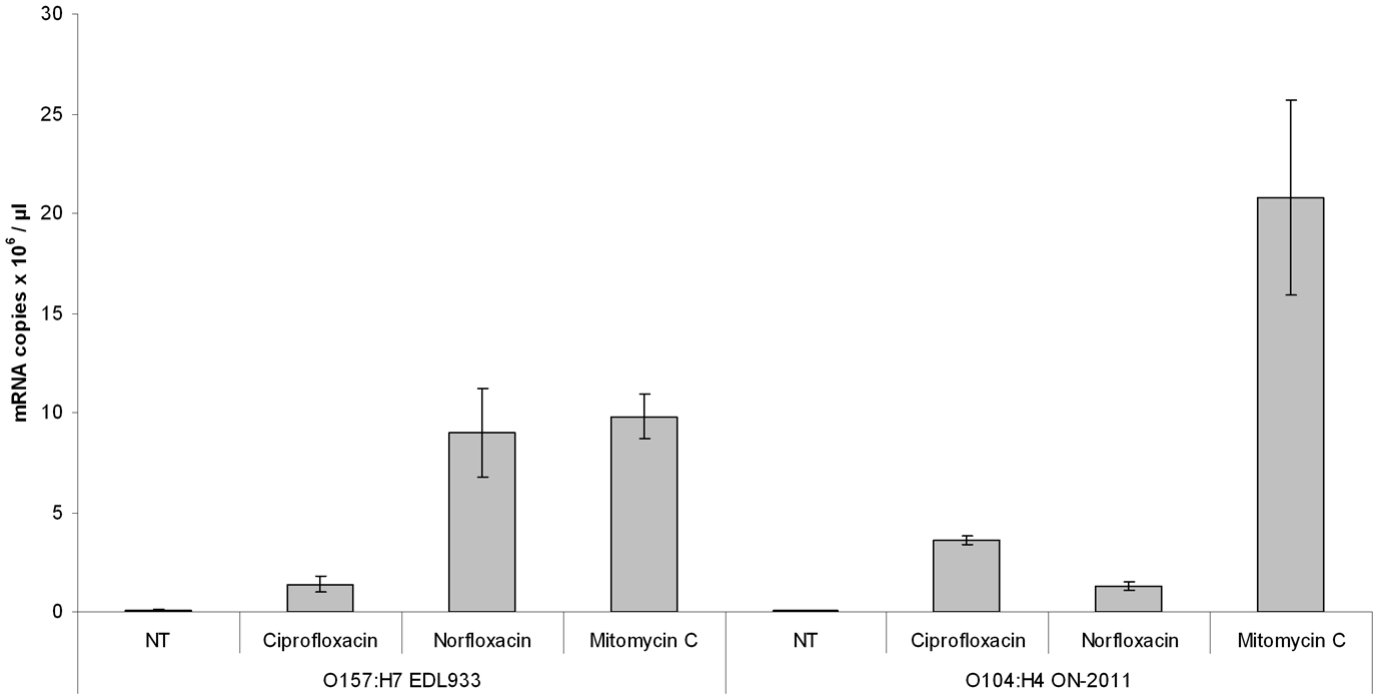
Stx2-mRNA copy number differences between *E. coli* O104:H4 outbreak-related strain ON-2011 and *E. coli* O157:H7 strain EDL933 under ciprofloxacin, norfloxacin and mitomycin C treatments. NT =  no treatment. Error bars represent standard deviations from three independent replicates.

Stx2-mRNA levels were also compared between O104:H4 strain ON-2011 and O157:H7 strain EDL933 after induction by ciprofloxacin, norfloxacin and mitomycin C (Figure 7). All three treatments increased Stx2-mRNA production in the strains, with the mitomycin C treatment causing the largest increase in Stx2-mRNA production by both strains. The amount of Stx2-mRNA produced by ON-2011 after mitomycin C treatment was significantly greater than that produced by EDL933 (P<0.05).

## Discussion

### Pan-genomic Comparison

The *E. coli* O104:H4 outbreak strain is thought to be derived from an *E. coli* O104:H4 progenitor that recently acquired the Stx2-phage [11]. Seven isolates of this outbreak strain were used in this study, but a detailed description of differences among these and other isolates can be found in the study by Grad et al. (2012) [24]. Stx-phage are temperate lambdoid phage, which exist as an ordered set of interchangeable modules that have a high propensity to recombine into novel mosaic configurations in the laboratory [25]. As Stx2-phage can be readily horizontally transferred, the evolutionary history of the phage and its bacterial host would not be expected to be highly concordant. We wished to identify the Stx2-phage most closely related to the *E. coli* O104:H4 outbreak Stx2-phage, by considering the bacteriophage sequence independently of that of the host bacteria.

Given the potential for high levels of recombination among Stx2-bacteriophage, we initially examined the phylogenetic relationship of Stx2-phage from 51 *E. coli* strains and *Shigella dysenteriae* using a network, where competing phylogenetic signals would be shown. We also chose to limit the Stx2-phage “pan-genome” to loci present in at least three strains, as loci present in two or fewer are typically not informative phylogenetically [26].

Brzuszkiewicz et al. [5] had previously found that the VT2phi_272 O157:H7 bacteriophage from *E. coli* strain 71074 was closely related to the Stx2-phage from the *E. coli* O104:H4 outbreak strain. This was particularly interesting to us, as strain 71074 is known to be from a clade of *E. coli* O157:H7 lineage I/II strains that has been termed “hyper-virulent” due to its more frequent association with severe human infections compared to other *E. coli* O157:H7 genotypes [27], [28]. However, it was unclear how many Stx2-phage sequences had been examined in the Brzuszkiewicz et al. [5] study, so we repeated the analysis using 51 Stx2-phage sequences and the outgroup *Shigella dysenteriae* phage sequence.

Both the neighbour-net and ML-tree examination of the pan-genomic data grouped the Stx2-phage from the O104:H4 outbreak isolates as a cluster, with the common closest phage being from O111:H- strain JB1-95. While the phage from O157:H7 lineage I/II strains bear a structural similarity to the phage from the O104:H4 outbreak strain, our study suggests the possibility of a lateral gene transfer event from an STEC host more closely related to JB1-95 than any of the O157:H7 strains.

### Evolutionary Concordance of Stx2-bacteriophage and Host-genomes

Despite the expectation that Stx2-phage would be highly heterogeneous, most were highly concordant with the phylogenetic trees of their bacterial hosts, suggesting that the host and phage had stably co-evolved for significant periods of time. This suggests that the lysogen offers an advantage to its host in the natural environment of the bacteria that is greater than the evolutionary cost of the bacterium continually copying the DNA of the foreign bacteriophage genome. The natural reservoir of most Stx2-producing bacteria is the ruminant intestine [29]; therefore, the evolutionary forces acting to select for particular traits are those that confer an advantage in the ruminant host, or the “source” habitat [30]. Humans are largely incidental hosts, with infection and clearance lasting only a few weeks and represent a “sink” habitat. In this source/sink evolutionary model, the source habitat selects for long-term evolutionary change and the sink habitat allows proliferation over a limited time-scale, with eventual clearance.

In the case of Stx, it may aid in the colonization of the gut [31] and has also been suggested to defend the population against grazing protozoa that inhabit the bovine intestinal tract [32]. This resistance to predation may also facilitate increased environmental persistence and re-uptake by ruminants, allowing a single-clone to dominate in a herd, as has been shown in *E. coli* O157:H7 [33], [34]. The increased environmental persistence if associated with toxin production would increase the risk of human infection by increasing the probability of exposure. Once infected, the amount of toxin produced may also help the pathogen survive the human immune response, as Stx production has been shown to allow proliferation of *E. coli* O157:H7 within human macrophages [35]. Additionally, immunity to predatory bacteriophages in the rumen gut could also select for the persistence of lysogenic Stx-bacteriophage in *E. coli* hosts.

We observed in the whole-genome tree of Figure 3 that serotypes invariably clustered together, suggesting that the discordance between the Stx2-phage and *E. coli* host whole genome trees was caused by recent acquisition (horizontal transfer) of the Stx2-phage. The evolution of pathogenicity in *E. coli* is thought to be driven by events such as phage-mediated horizontal gene transfer and our results suggest that once a phage has been acquired, it can be stably propagated along with the bacterial lineage [36]. This is most evident in the case of the O157:H7 strains, where the whole-genomic sequences in Figure 3 are clearly distributed among the three lineages, and the Stx2-phage also group according to lineage (Figures 1 and 2), even in the case where a single strain has two integrated Stx2-phage (Stx2 and Stx2c). The grouping of non-O157:H7 Stx2-phage with O157:H7 Stx2-phage on the same branch of the tree, as was the case for the O145:H2 strain and O157:H7 lineage I Stx2-phage, likely indicates horizontal transfer of the phage between organisms. In the case of the O111 Stx2-bacteriophage, their wider distribution among STEC strains also suggests at least one horizontal transfer event.

### Stx2-bacteriophage Sequence Alignment

*E. coli* O111:H- strain JB1-95 was found to be more closely related to the *E. coli* O104:H4 outbreak isolates than the previously reported Stx2-phage from O157:H7 lineage I/II strain 71074 (VT2phi_272) and among the other Stx2-phage sequences examined.

A plausible Stx2-phage evolutionary scenario involves an STEC O111:H- like common ancestor to O111:H- JB1-95 and the *E. coli* O104:H4 outbreak isolates undergoing a lateral transfer event to the O104:H4 outbreak progenitor strain. This would account for the similar evolutionary histories of the Stx2-phage, as well as account for the region missing from the JB1-95 Stx2-phage sequence but present in all other closely related strains, which seems to indicate a loss of genetic information in the O111:H- branch. However, it should be noted that some Stx2-phage sequences were incompletely recovered from the draft whole-genome sequence as a result of the Stx2-phage sequence being distributed among multiple contigs or the absence of Stx2-phage sequence from the draft-genome. Assembled genome sequences for all of these strains would be helpful in more precisely defining these relationships. The proposed evolutionary history of the *E. coli* O104:H4 bacterial ancestor giving rise to the 2011 *E. coli* O104:H4 Stx2-containing strain and EAEC strain 55989 has recently been presented by Mellmann et al. [11].

Stx2-phage are known to have an integration site preference in host bacteria [37]. Because this integration is dependent on the host genome, the site occupied by an integrated Stx2-phage can be used to discriminate among members of the same species [38]. The fact that the Stx2-bacteriophage in the O104:H4 outbreak isolates, the *E. coli* O157:H7 lineage I/II strains and the O111:H- strains are all inserted in the *argW* locus offers additional evidence that these bacteriophage are closely related. Other *E. coli* strains within serotypes O111:H- and O157:H7 possess Stx2-phage that are significantly different from those of the *E. coli* O104:H4 outbreak isolates, so we conclude that the Stx2-phage has been introduced multiple times within STEC through horizontal transfer.

The parallel evolution of this and other STEC virulence factors has been previously described [39]. For example, evidence suggests the locus of enterocyte effacement (LEE) has been obtained through horizontal transfer multiple times in *E. coli*, giving rise to two distinct groupings of Stx-negative enteropathogenic *E. coli* (EPEC) and Stx-positive enterohemorrhagic *E. coli* (EHEC) strains that share a LEE insertion site [40]. EPEC typically cause diarrhea in humans, while EHEC cause hemorrhagic colitis and HUS. These findings and the results of this study support the idea that multiple genomic backgrounds in *E. coli* are able to persist in the human gastrointestinal tract, and in some cases cause illness through a variety of distinct mechanisms; however, it is the Shiga toxin, and in particular Stx2 that is responsible for severe forms of human disease such as HUS. It is now clear that Stx2-phage are capable of integrating into diverse *E. coli* genomic backgrounds and of qualitatively transforming health risks associated with *E. coli* strains from other pathogroups such as EPEC and EAEC.

### Stx2-toxin Production

*E. coli* O157:H7 clade 8 lineage I/II strains are more frequently associated with HUS than other *E. coli* O157 genotypes and the incidence of human infection with clade 8 strains is thought to be increasing [28]. It is interesting to note that the Stx2-phage from these “hyper-virulent” *E. coli* O157 strains bear a striking structural similarity to the Stx2-phage from the *E. coli* O104:H4 outbreak isolates. The *E. coli* O104:H4 outbreak isolates are also associated with high rates of HUS, suggesting that these phage share attributes that contribute to increased virulence through mechanisms such as increased expression of Stx2. We have previously shown that lineage I strains of *E. coli* O157:H7 produce significantly different amounts of Stx2 in vitro than lineage II strains, and that within lineage I, strains from humans produce significantly more Stx2 than strains from cattle [41]. In the current study, we found that an *E. coli* O104:H4 outbreak-related isolate (ON-2011) produced significantly more Stx2 and Stx2-mRNA than outbreak-related lineage I *E. coli* O157:H7 strains after mitomycin C induction. However, without mitomycin C added to the culture, O104:H4 strain ON-2011 produced very little toxin. Stx2 is an extra-cellular toxin and has been shown to be released from STEC via two specific mechanisms [42]. Therefore, it is unlikely that the high level of Stx2 produced by the mitomycin C-treated *E. coli* O104 strain was the result of differential toxin release from cell membranes by polymyxin B; however, this possibility cannot be excluded without further study.

A recent study examining the effect of antimicrobials on isolates from the German O104:H4 outbreak found that ciprofloxacin significantly increased both Stx2 and Stx2-mRNA production [43], which corroborates our current findings and suggests that the Stx2-phage from the *E. coli* O104:H4 outbreak strains is induced and undergoes lytic conversion *in vivo* in a significant proportion of the bacterial population in the presence of both ciprofloxacin and mitomycin C.

While it can be argued that conditions in the gastrointestinal tract could induce a similar response to that obtained with mitomycin C, further study is required to determine if Stx2 production by *E. coli* O104:H4 is greater or lesser than for *E. coli* O157 strains *in vivo*, particularly since other work has shown down-regulation of toxin-production by *E. coli* O157:H7 strains due to microbiota secreted factors present in the human gut [44].

It is also unknown whether the high-prevalence of serious human disease caused by the O104:H4 outbreak isolates and other Stx2-producing bacteria is due simply to human exposure to high-numbers of the organism, or whether the production of high-levels of Stx2 are responsible for the high-levels of serious human disease. Credence is given to the first hypothesis by the “supershedder” phenomenon in cattle, where it has been reported that phage type (PT) 21/28 of *E. coli* O157:H7 is shed in significantly higher numbers by cattle than other PTs, and PT 21/28 is also the most commonly associated with serious human disease, suggesting that higher levels of human exposure to this PT account for its increased association with human disease [33]. Conversely, it may be that certain strains are more virulent in humans, simply by virtue of the amount of toxin or other virulence factors they produce. In theory, it would only take one such organism to establish and proliferate within the human intestine to cause severe human illness. Factors allowing the bacteria to withstand the hardships of the human gut and immune system, such as acid resistance and toxin-production could significantly influence disease outcomes. We have shown that in cultures containing mitomycin C, an O104:H4 strain produced significantly higher levels of Stx2 than *E. coli* O157:H7 strains. It was also recently shown that O104:H4 outbreak isolates also had a higher number of cells survive pH 2.5 conditions than *E. coli* O157:H7 strain EDL933 [11].

It has also been suggested that the increased virulence of the *E. coli* O104:H4 outbreak strain could in part be related to the EAEC bacterial host genome, which has evolved adaptations for attachment and survival in the human intestine and that these adaptations have facilitated the systemic absorption of Stx2, which in turn increased the risk of developing HUS [3], [45]. If true, it is likely that the acquisition of any one of the many Stx2-phage would have led to an increase in virulence of the *E. coli* O104:H4 host. However, the relative importance of Stx2-phage heterogeneity and specific Stx2-phage features compared to those of the bacterial host in the virulence of the *E. coli* O104:H4 outbreak strain awaits further study.

### Conclusions

It is clear from the German *E. coli* O104:H4 outbreak that future novel combinations of bacteriophage and bacterial host are likely and that their impact on human health could be devastating. According to the source-sink model, attributes such as Stx production by STEC from ruminants is thought to provide a selective advantage in the animal reservoir, not in the “dead end” human host. STEC virulence in humans is therefore not selected for but is an unintended consequence of accidental infection. As EAEC strains are thought to be human-restricted, the acquisition of a Stx2-phage from an O111:H- like strain had to occur in an environment where both EAEC and STEC or their phage were concurrently present. This phenotypic jump could have been in the intestine of an animal or human harbouring both *E. coli* pathotypes, or an environment where both human and ruminant feces were present. In the case of the *E. coli* O104:H4 STEC strain, we do not know if the acquisition of this Stx2-phage has provided a selective advantage for the bacterium in humans. While the infection was clearly foodborne, it would appear that human to human transmission has not been sustained and no new cases have been reported. The findings of this study suggest that efforts to control all *E. coli* pathogroups associated with enteric disease in both developed and developing countries as well as Stx2-phage from both human and animal sources are warranted to prevent the re-occurrence of similar devastating outbreaks.

## Supporting Information

Figure S1 **The neighbour-net visualizations of the Stx2-phage “pan-genome” using fragmentation sizes from 50 bp to 1000 bp at both an 85% and a 95% sequence identity threshold.** The number to isolate name correspondence is as follows: 1 = O147 UMNF18, 2 = O104:H4 ON211, 3 = O104:H4 CS70, 4 = O157:H7 EC4076, 5 = O157:H7 EC970520, 6 = O103:H2 12009, 7 = O157:H7 EC4206, 8 = Bacteriophage stx2_I, 9 = O157:H7 71074, 10 = O104:H4 C227-11, 11 = O104:H4 GOS2, 12 = O157:H7 TW14588_2, 13 = O121:H19 5.0959, 14 = O139 S1191 stx2e, 15 = O157:H7 EC869, 16 = O157:H7 EC4196, 17 = O157:H7 EC4115, 18 = O104:H4 GOS1, 19 = O111:H- 11128, 20 = O157:H7 EC1212, 21 = Shigella dysenteriae Sd197 stx1, 22 = O157:H7 EC4115, 23 = O157:H7 EC4486, 24 = O153 3.3884, 25 = O157:H7 LRH6, 26 = O104:H4 H112180540 CS110, 27 = O157:H7 EC4206, 28 = O157:H7 1044, 29 = O157:H7 EC4113, 30 = O147:H- 2.3916 stx2e, 31 = Bacteriophage 2851, 32 = Bacteriophage stx2_II, 33 = O157:H7 Sakai, 34 = O111:H- JB1-95, 35 = O157:H7 EC4024, 36 = O157:H7 EDL933, 37 = O157:H7 TW14588_1, 38 = Ont:H12 EH250 stx2d, 39 = O157:H7 TW14359, 40 = O104:H4 TY-2482, 41 = O111:NM OK1180, 42 = Bacteriophage 933****W, 43 = O157:H7 EC4042, 44 = O157:H7 TW14359, 45 = O145:H2 4.0967, 46 = O157:H7 EC508, 47 = O91:H21 B2F1, 48 = O73:H16 C165-02 stx2d, 49 = O157:H7 1125 ECF, 50 = O157:H7 EC4042, 51 = O157:H7 EC4401, 52 = O128:H2 9.0111.
(TIF)

Dataset S1 **The MAFFT sequence alignment of the complete Stx2-phage genomes used for the ML tree of** **Figure 5** **in Phylip format.**
(PHY)
