# Supplementary figures and images for: A Comparison of Shiga-Toxin 2 Bacteriophage from Classical Enterohemorrhagic Escherichia coli Serotypes and the German E. coli O104:H4 Outbreak Strain
Source: PLoS One. 2012 May 23;7(5):e37362. doi: 10.1371/journal.pone.0037362 (PMC3359367; doi:10.1371/journal.pone.0037362)

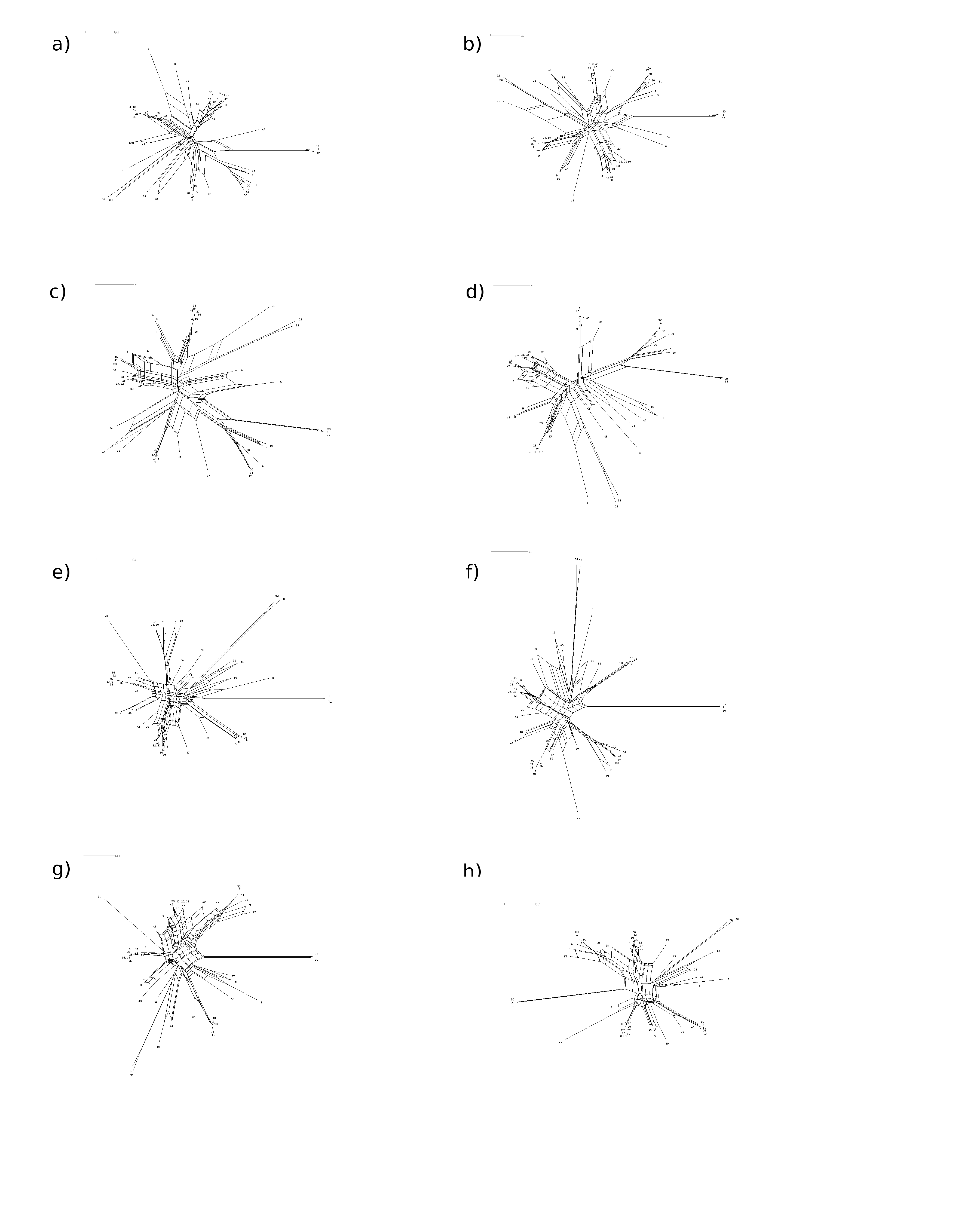

Supplement: Figure S1 — The neighbour-net visualizations of the Stx2-phage “pan-genome” using fragmentation sizes from 50 bp to 1000 bp at both an 85% and a 95% sequence identity threshold. The number to isolate name correspondence is as follows: 1 = O147 UMNF18, 2 = O104:H4 ON211, 3 = O104:H4 CS70, 4 = O157:H7 EC4076, 5 = O157:H7 EC970520, 6 = O103:H2 12009, 7 = O157:H7 EC4206, 8 = Bacteriophage stx2_I, 9 = O157:H7 71074, 10 = O104:H4 C227-11, 11 = O104:H4 GOS2, 12 = O157:H7 TW14588_2, 13 = O121:H19 5.0959, 14 = O139 S1191 stx2e, 15 = O157:H7 EC869, 16 = O157:H7 EC4196, 17 = O157:H7 EC4115, 18 = O104:H4 GOS1, 19 = O111:H- 11128, 20 = O157:H7 EC1212, 21 = Shigella dysenteriae Sd197 stx1, 22 = O157:H7 EC4115, 23 = O157:H7 EC4486, 24 = O153 3.3884, 25 = O157:H7 LRH6, 26 = O104:H4 H112180540 CS110, 27 = O157:H7 EC4206, 28 = O157:H7 1044, 29 = O157:H7 EC4113, 30 = O147:H- 2.3916 stx2e, 31 = Bacteriophage 2851, 32 = Bacteriophage stx2_II, 33 = O157:H7 Sakai, 34 = O111:H- JB1-95, 35 = O157:H7 EC4024, 36 = O157:H7 EDL933, 37 = O157:H7 TW14588_1, 38 = Ont:H12 EH250 stx2d, 39 = O157:H7 TW14359, 40 = O104:H4 TY-2482, 41 = O111:NM OK1180, 42 = Bacteriophage 933W, 43 = O157:H7 EC4042, 44 = O157:H7 TW14359, 45 = O145:H2 4.0967, 46 = O157:H7 EC508, 47 = O91:H21 B2F1, 48 = O73:H16 C165-02 stx2d, 49 = O157:H7 1125 ECF, 50 = O157:H7 EC4042, 51 = O157:H7 EC4401, 52 = O128:H2 9.0111. (TIF) [file pone.0037362.s001.tif]
